# Transient Asymptomatic Sinus Bradycardia and Sinus Pauses with Bevacizumab: Case Report and Literature Review

**DOI:** 10.7759/cureus.6177

**Published:** 2019-11-18

**Authors:** Amr Essa, Osama Diab, Ahmed Munir, Venkata Andukuri

**Affiliations:** 1 Internal Medicine, Creighton University School of Medicine, Omaha, USA; 2 Hematology and Oncology, Kansas University Medical Center, Kansas, USA; 3 Pulmonary/Critical Care Medicine, State University of New York, Buffalo, USA; 4 Internal Medicine, Creighton University Medical Center, Omaha, USA

**Keywords:** bevacizumab, anti-vascular endothelial growth factor (anti-vegf), urothelial carcinoma, sinus pause, targeted therapy

## Abstract

Systemic side effects of anti-cancer therapy remain a major limiting factor for patients, even with targeted therapy. Bevacizumab is an example of targeted cancer therapy which targets the vascular endothelial growth factor receptor (VEGFR) that has been approved for the treatment of various cancers and has been evaluated in metastatic urothelial carcinoma (MUC). We report a case of MUC on bevacizumab containing regimen who developed temporary asymptomatic sinus bradycardia with sinus pauses. That adverse event was thought to be related to the bevacizumab in her cancer regimen. Her Holter monitoring recording for a total duration of 28 days and 14 h after discharge did not show recurrence of sinus pauses. This case indicates the necessity for observation for the cardiac conduction defects as side effects in patients receiving bevacizumab, especially since they might be asymptomatic and transient.

## Introduction

The mechanism of traditional chemotherapy involves targeting nonspecific, rapidly dividing cells, including noncancerous body cells, which often lead to significant side effects. After developing a better understanding of the regulatory signals and functioning process of tumor cells on a molecular level, new mechanisms of targeting specific signals and carcinogens have been developed [1]. That led to the development of targeted cancer therapy. Monoclonal antibodies against tumor angiogenic molecules represent a fundamental example of targeted cancer therapy. Bevacizumab is an example of a recombinant humanized monoclonal anti-vascular endothelial growth factor (VEGF) antibody developed to bind VEGF blocking the VEGF/vascular endothelial growth factor receptor (VEGFR) interaction [2] and it prevents angiogenesis which is a vital step for tumor growth, invasion, and spread. Bevacizumab-associated cardiovascular side effects have been reported in the literature, and most of them are hypertension (HTN), congestive heart failure (CHF), and thromboembolism [3]. However, bioelectrical dysfunction and arrhythmia associated with bevacizumab are rare. Here we present a case of asymptomatic transient sinus bradycardia with sinus pauses thought to be linked to bevacizumab. 

## Case presentation

A 61-year-old female with a history of recurrent metastatic urothelial carcinoma (MUC) on atezolizumab/bevacizumab regimen and hypothyroidism with no past cardiac history presented with fatigue, polyuria, and polydipsia.

**Figure 1:**
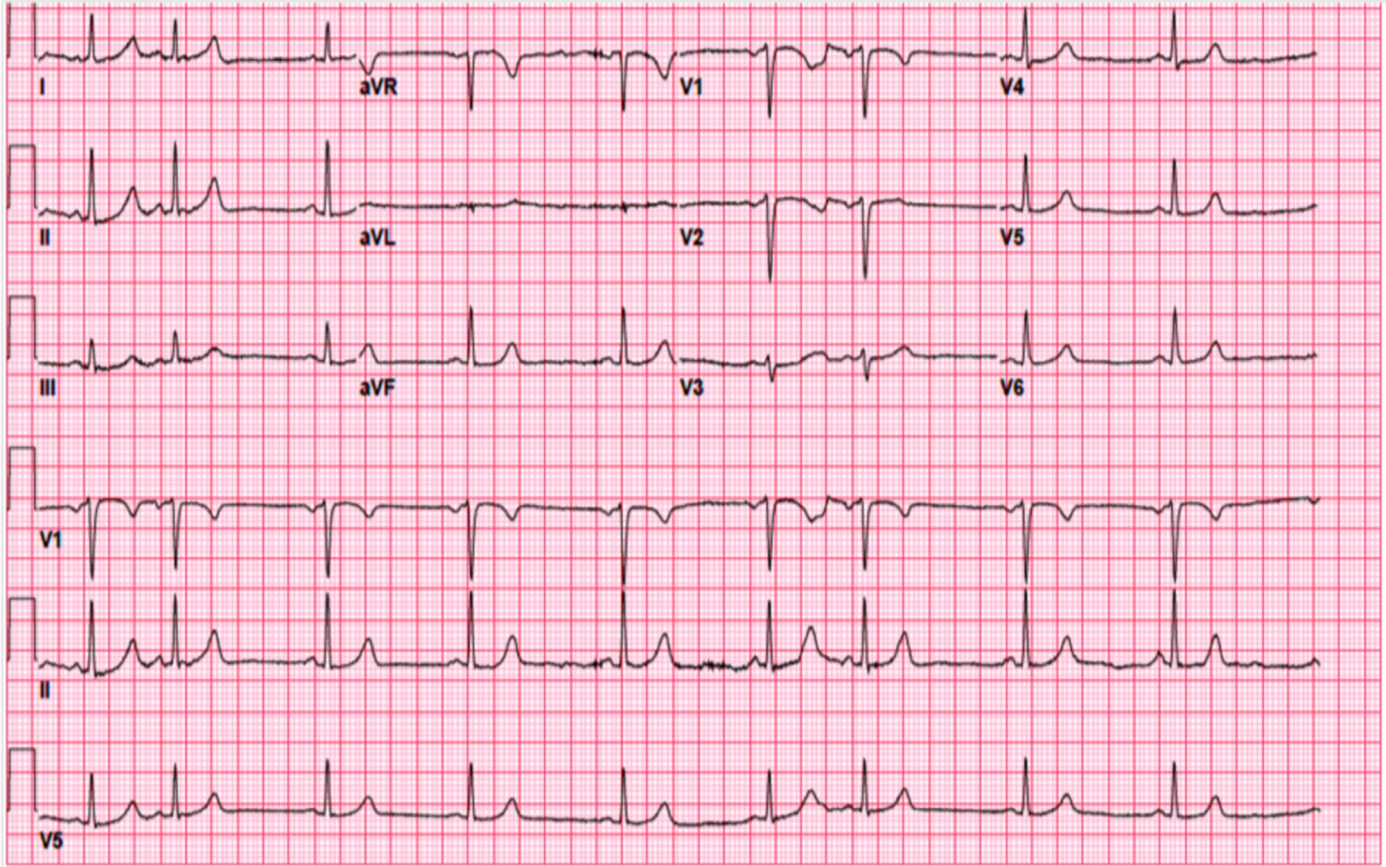
EKG on the presentation. EKG, electrocardiogram

On presentation, blood work-up showed hyperglycemia, hyperkalemia, elevated serum creatinine, high anion-gap, high B-hydroxybutyric acid, mild anemia, and mild thrombocytopenia. The rest of the basic metabolic panel and electrolytes were unremarkable. Complete blood count showed mild anemia and thrombocytopenia (Table 1). Electrocardiogram (EKG) on admission showed sinus bradycardia with premature atrial complexes with a heart rate of 57 beats/minute with normal PR interval (Figure 1). 

**Table 1:** Lab work up on the presentation. TSH, thyroid stimulating hormone

	Value	Normal value
Sodium	122 mmol/L	135-145 mmol/L
Potassium	5.7 mmol/L	3.7-5.1 mmol/L
Chloride	91 mmol/L	96-1100 mmol/L
Carbon dioxide	15 mmol/L	22-31 mmol/L
Blood urea nitrogen	43 mg/dL	6-24 mg/dL
Creatinine	1.9 mg/dL	0.50-1.10 mg/dL
Calcium	9.2 mg/dL	8.5-10.5 mg/dL
Magnesium	2 mg/dL	1.3-2.6 mg/dL
Phosphorus	4.9 mg/dL	2.5-4.9 mg/dL
Glucose	1045 mg/dL	70-100 mg/dL
Beta-hydroxybutyrate	53.3 mg/dL	0.2-2.8 mg/dL
Serum osmolarity	321 mOsm/kg	285-305 mOsm/kg
Hemoglobin	10.3 gm/dL	12-16 gm/dL
White blood cell	7.3 k/uL	4-12 k/uL
Platelets	133 k/uL	140-440 ku/uL
Hemoglobin A1C	7.6%	4.5%-5.6%
TSH	6.060 UIU/mL	0.400-3.800 UIU/mL
Free thyroxin (T4, free)	1.4 ng/dL	0.7-1.4 ng/dL
Free tri-iodothyronine (T3, free)	0.9 pg/mL	2.1-3.8 pg/mL

The patient was started on treatment for diabetic ketoacidosis (DKA) per protocol with intravenous fluid and insulin per protocol steady improvement in her clinical status and lab values the following day. During her hospital stay, she exhibited asymptomatic sinus bradycardia on telemetry with a heart rate of 35-50 beats per minute and frequent sinus pauses of different durations: 2.3, 2.8, 3.4 s. Repeat EKG showed sinus bradycardia with a heart rate of 56 beats/minute and no other abnormalities. 

**Figure 2:**
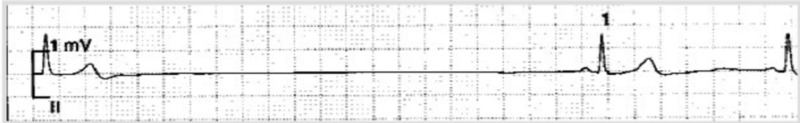
Telemetry strip showing 4.26 s sinus pause.

Transthoracic echocardiogram (TTE) revealed normal left ventricular ejection fraction of 60%-65% with normal wall motion. Troponin was checked and was negative on three occasions. Her thyroid function was tested, and her levothyroxine dose was up titrated as her thyroid stimulating hormone (TSH) was elevated with normal free T4. Her electrolytes were being replaced daily. She continued to have sinus bradycardia, and frequent sinus pauses on the telemetry with the longest pause of 4.26 s (Figure 2) on day 4 of the hospital stay. However, she stayed asymptomatic during her entire hospital stay.

**Figure 3:**
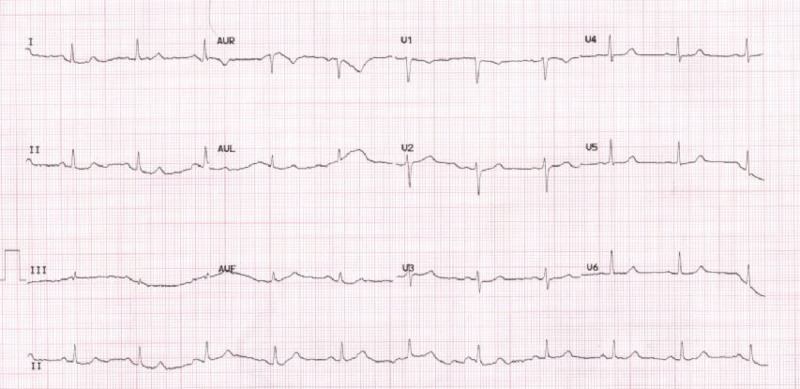
Normal EKG six years before presentation. EKG, electrocardiogram

**Figure 4:**
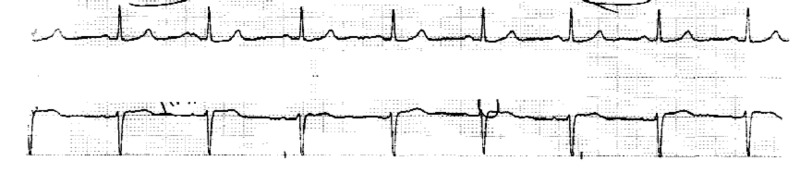
Normal telemetry strip three years before presentation.

The patient was not on any atrial-ventricular (AV) nodal blocking agents or medications that can cause bradycardia. No evident causes of her sinus bradycardia with sinus pauses could be identified. The electrophysiology cardiology service was consulted, and their recommendations suggested no indication for pacemaker placement as her findings were thought to be related to her anti-cancer therapy. She was on atezolizumab 1200 mg/kg/IV and bevacizumab 15 mg/kg/IV every three weeks. Bevacizumab has been into her anti-cancer regimen for seven months before the admission, and the last dose was two weeks before the admission. No changes were made to her anti-cancer regimen on discharge. Reviewing her old available records did not show any history of sinus bradycardia or sinus pauses. One EKG dated six years before the presentation was reviewed and showed normal sinus rhythm with no abnormality (Figure 3). Also, an available telemetry strip dated three years before the presentation was reviewed and showed normal sinus rhythm with no arrhythmia or pauses (Figure 4).

**Figure 5:**
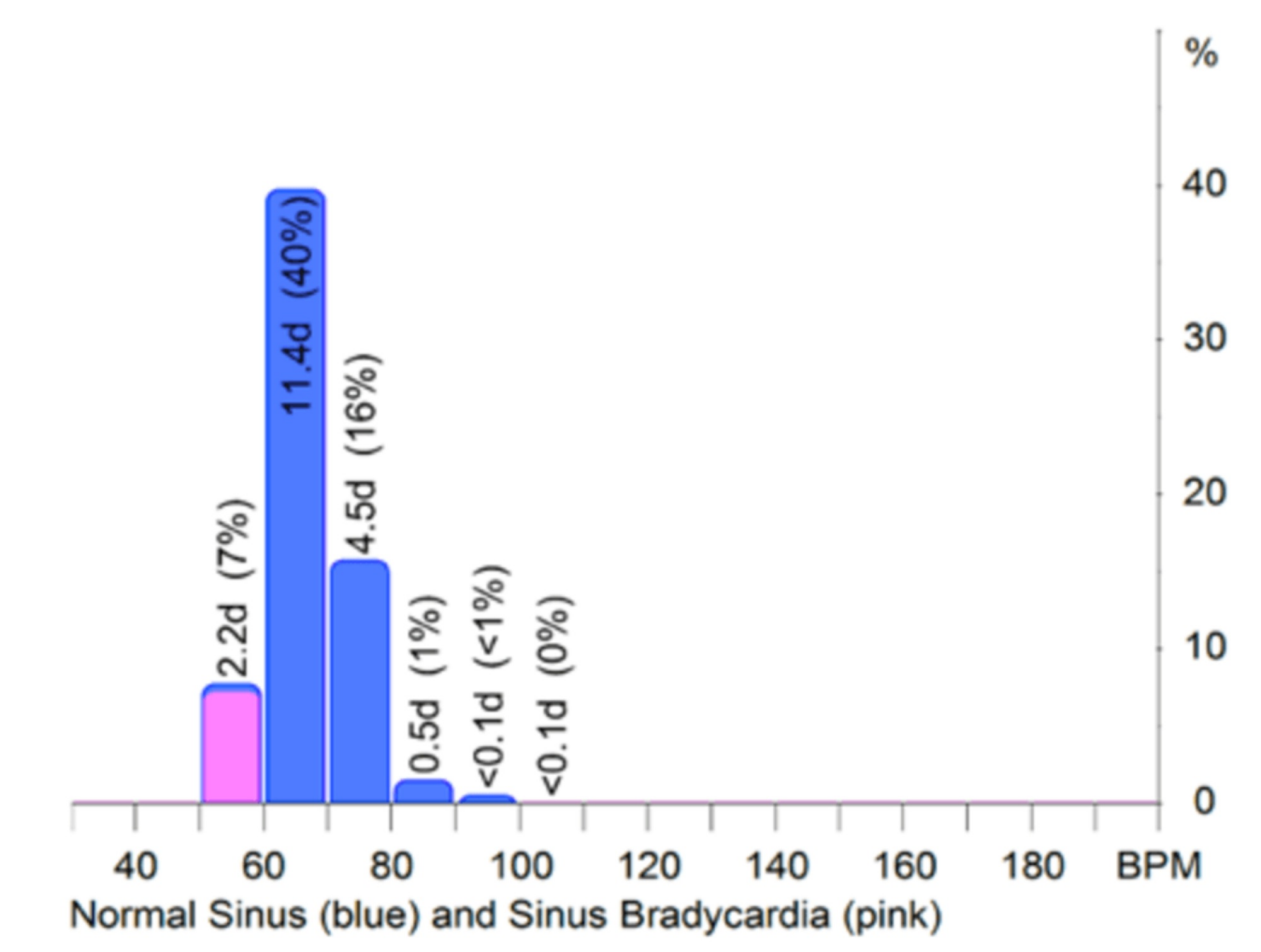
Holter monitoring report after discharge.

The patient was discharged after eight days of admission with a holter monitor for a total duration of 28 days and 14 h after discharge, which revealed the patient's primary rhythm to be in normal sinus rhythm. The average heart rate was 65 beats/minute. Approximately 9% of the time was spent in sinus bradycardia, and there was no recurrence of sinus pauses episodes (Figure 5). 

## Discussion

Angiogenesis is regulated through an interaction between angiogenic molecules and their receptors, and VEGF is an important example of angiogenic molecules. The interaction between VEGF and its tyrosine kinase receptor, VEGF receptor (VEGFR), signal initiation of multiple molecular pathways are critical for endothelial survival and proliferation [4]. Bevacizumab is an example of a recombinant humanized monoclonal anti-VEGF antibody developed to bind VEGF blocking the VEGF/VEGFR [2] and it prevents angiogenesis. VEGF/VEGR interaction plays an essential role in the pathophysiology of urothelial cancer and is expressed in 50% of urothelial carcinomas [5]. Hence bevacizumab has been evaluated as first-line therapy for MUC [6-8] and in neoadjuvant settings [9-10].

Cardiovascular side effects secondary to anti-cancer therapy can be either temporary or permanent, idiosyncratic, or dose-related. Other effects could be potentiated by combining multiple anti-cancer drugs in the regimen. With bevacizumab, HTN side effects appear to be dose-dependent, and its incidence augmented by combining with other anti-cancer therapy. CHF side effects appear to be increased in patients with prior exposure to anthracyclines, baseline cardiomyopathy, or prior chest wall irradiation. Arterial thromboembolism side effect also appears to be augmented by combining bevacizumab with other anti-cancer agents. Venous thromboembolism appears to be related to the dose of bevacizumab. The precise mechanism of cardiovascular side effects associated with bevacizumab is unknown. However, it is thought to be related to the VEGF role in vasculature protection, inhibition of vascular smooth muscle proliferation, upregulation of endothelial nitric oxide synthase, and inhibition of platelet aggregation [3].

The incidence of bradycardia associated with bevacizumab is rare. However, it has been reported in clinical trials. Sinus bradycardia was reported in 3% of patients in a phase II study of bevacizumab and vorinostat for patients with Grade 4 malignant glioma [11]. Grade 3 sinus bradycardia was reported in 1.6% of patients in Phase II study of cisplatin plus etoposide and bevacizumab for previously untreated, extensive-stage small-cell lung cancer [12]. Grade 3 sinus bradycardia was reported in one patient who received axitinib combined with FOLFOX plus bevacizumab regimen in a Phase I study of axitinib in combination with bevacizumab plus chemotherapy or chemotherapy alone in patients with metastatic colorectal cancer and other solid tumors [13]. Bradycardia was associated with the group of patients treated with bevacizumab containing regimen in a study to evaluate the efficacy of bevacizumab in a combination of irinotecan, fluorouracil, and leucovorin for metastatic colorectal cancer treated by failed prior oxaliplatin-based regimen [14].

In a literature search, only two cases have linked sinus bradycardia to bevacizumab. Zekri [15] described temporary asymptotic sinus bradycardia in a patient with non-small cell lung cancer during an infusion of paclitaxel, carboplatin, and bevacizumab (PCB) regimen. That adverse reaction was linked to paclitaxel in the regimen, which has a well-documented incidence of bradycardia as a side effect [16]. In another report, Mori et al. [17] described temporary sinus bradycardia in a patient with metastatic colon cancer on the 5-FU/leucovorin/oxaliplatin (FOLFOX)/bevacizumab regimen. However, the temporary sinus bradycardia was associated with an infusion reaction to the bevacizumab infusion and started 5 h after the infusion reaction. In a third report, Chino et al. described cardiogenic syncope secondary to left ventricular dysfunction, not arrhythmia-related in a nonsmall cell lung cancer patient [18]. 

Although atezolizumab has no documented adverse effect of bradycardia based on the most recent, largest, and most comprehensive meta-analysis of treatment-related adverse events for immune checkpoint inhibitors [19], a synergistic or potentiated effect due to the combination therapy cannot be excluded.

For our patient, there was no prior cardiac history and no history of sinus bradycardia or sinus pauses events in her old records. We thought her sinus bradycardia with sinus pauses was related to bevacizumab with no other evidence of an alternative explanation. The patient had no clinical symptoms related to that adverse effect, and it was transient; moreover, the timeline of the events suggests a possibility of a latent period before the development of the bioelectrical cardiac side effect.

## Conclusions

Although targeting more specific cellular pathways and molecules in targeted cancer therapy are associated with fewer adverse effects, systemic side effects are still a limiting factor for these agents with its emerging application in clinical practice. In patients receiving bevacizumab, cardiac bioelectric dysfunction such as bradycardia can be easily missed in the outpatient setting as the onset is not immediate, and patients may remain asymptomatic. With the increasing utilization of bevacizumab in the clinical world, we recommend further studies in order to better understand its full profile and mechanism in the development of cardiovascular side effects. 
